# Strengthening community engagement in the fight against hepatitis B in two regions of Cameroon

**DOI:** 10.4102/jphia.v16i1.1268

**Published:** 2025-09-05

**Authors:** Solange Neh Manju Atah, Nadege Goumkwa Mafopa, Cindy Lobe, Juliette-Laure N. Ndzie Ondigui, Jude S.Y. Atah, Joseph Nelson Siewe Fodjo, Patrick Awoumou, Alliance-Laure Otam, Martin N.D. Mokake, Puinta Peyonga, Rosi García Martinez Peñalver, Isabel Fernández Escobar, Desire Akaba, Felix Assah, Robinson Mbu Enow, Judith N. Torimiro

**Affiliations:** 1 Department of Public Health, Faculty of Medicine and Biomedical Sciences, University of Yaoundé 1, Yaoundé, Cameroon; 2 Department of Molecular Biology, Chantal BIYA International Reference Center for Research on HIV/AIDS Prevention and Management (CIRCB), Yaoundé, Cameroon; 3 Department of Biomedical Sciences, Faculty of Sciences, University of Ngaoundere, Ngaoundere, Cameroon; 4 Department of Microbiology, Faculty of Sciences, University of Yaoundé 1, Yaoundé, Cameroon; 5 Chantal BIYA International Reference Center for Research on HIV/AIDS Prevention and Management (CIRCB), Yaoundé, Cameroon; 6 Ayos Health District, Komdombo Medicalised Health Centre, Ayos, Cameroon; 7 Brain Research Africa Initiative (BRAIN), Yaoundé, Cameroon; 8 Department of Biochemistry, Faculty of Medicine and Biomedical Sciences, University of Yaoundé 1, Yaoundé, Cameroon; 9 Buea Regional Hospital Annex, Buea, Cameroon; 10 Bikop Catholic Health Center, Bikop, Cameroon; 11 Department of Anatomy, Faculty of Medicine and Biomedical Sciences, University of Yaoundé 1, Yaoundé, Cameroon; 12 Department of Gynecology and Obstetrics, Yaoundé Gynecology, Obstetrics and Pediatrics Hospital, Yaoundé, Cameroon

**Keywords:** hepatitis B, community, sensitisation, testing, counselling

## Abstract

**Background:**

In Cameroon, the prevalence of hepatitis B in the general population is 10%, and the vaccination coverage is less than 15% among adults. Although 80% have heard about the disease, only 50% know the modes of transmission and prevention of hepatitis B virus (HBV).

**Aim:**

To assess the knowledge, attitude and practice (KAP), sensitise the population and identify new cases of hepatitis B.

**Setting:**

Rural and urban areas of the Centre and South West Regions in Cameroon.

**Methods:**

An observational cross-sectional study was carried out from 2021 to 2023 on the general population to determine the HBV, seroprevalence and assess awareness of hepatitis B. We used modified Bloom’s cutoff to define KAP categories.

**Results:**

Seven hundred and fifty-nine and 456 persons gave consent to participate in the KAP and sero-surveys, respectively. About 20.4% (*n* = 155/759) of participants had never heard of hepatitis B. Among the 604 participants who had heard, 52.2% (*n* = 315/604) did not know at least one transmission route. However, 56.8% (*n* = 343/604) knew the hepatitis B vaccine, yet the vaccination coverage was 5.1% (*n* = 39/759). Furthermore, 69.5% (*n* = 420/604) had been tested at least once, 71.0% (*n* = 429/604) had inadequate knowledge and 68.7% (*n* = 415/604) had unfavourable practices. An association was observed between knowledge and practice, with an odds ratio of 4.1. HBV seroprevalence was 8.3%.

**Conclusion:**

Poor knowledge and lack of access to reliable information enhance the spread of hepatitis B. This propagation could be mitigated through sensitisation, voluntary counselling and testing (VCT) to identify new cases.

**Contribution:**

Highlights community-engaging initiatives to sensitise, test, prevent and treat hepatitis B.

## Background

Hepatitis B infection is a leading cause of hepatocellular carcinoma (HCC) and death from liver-related diseases globally.^1^ Of over 2 billion infected people globally, 296 million have chronic infections and 820 000 annual deaths have been recorded from liver-related diseases.^2^ Most chronic hepatitis B-infected adults were born during the pre-hepatitis-vaccination epoch. Despite the availability of efficacious vaccines nowadays, about 1.5 million persons still get infected yearly with hepatitis B.^3^ According to the World Health Organization (WHO), there are over 80 million hepatitis B-infected persons in sub-Saharan Africa, and 370 000 newborns get infected annually in this region.^3^ Hepatitis B is caused by the hepatitis B virus (HBV), which is vertically (mother-to-child) and horizontally (unprotected sexual intercourse, blood transfusion, use of contaminated sharp objects) transmitted. Although sub-Saharan Africa is a highly endemic zone of hepatitis B, a majority of the population is unaware of the disease, its mode of transmission and prevention.^4^ This lack of awareness of the disease can further promote the spread of hepatitis B infection on the continent through its principal modes of transmission. To curb the problem of HBV transmission, vaccination has been recommended as the mainstay method of prevention. In 1992, WHO recommended the incorporation of the hepatitis B vaccine in the Expanded Program on Immunisation (EPI), but the implementation was not immediate in many sub-Saharan African countries.^3^ Generally, while vaccines are available for infants at no cost, they are less affordable and accessible to adults. Limited accessibility will substantially determine vaccination coverage rates among the general adult population. In Cameroon, just like in other sub-Saharan countries, the awareness of hepatitis B is generally low.^4^ This current state of affairs makes immunisation as well as sensitisation on the disease primordial. However, the hepatitis B vaccine was incorporated into the national EPI of the country in 2005. The adult population of the hepatitis B pre-vaccination era has a relatively low vaccination coverage, even among the sexually active and other populations at risk, thereby increasing the spread of the disease.^5^ Cameroon, like other countries, seeks to eliminate hepatitis B by 2030 as per the WHO goals. There is no existing structured national programme tailored towards the fight against the disease. However, strategies put in place to eliminate the disease principally focus on vaccination, awareness and mass screening as well as increased community engagement. Emphasis on the urgency to control the disease and reduce its public health burden points to the WHO elimination goals in the Global Hepatitis Health Sector Strategy (GHSS) to eliminate viral hepatitis by 2030 (aimed at reducing to < 0.1% the prevalence of hepatitis B surface antigen [HBsAg] in children under 5 years and reduce by 90% the number of new cases, 65% the number of HBV-related deaths and to treat 80% of eligible persons infected with HBV).^5^

Vaccination is a mainstay intervention against vertical (when administered within 24 h of life) and horizontal transmission.^3^ Therefore, creating awareness of the disease, its modes of transmission and prevention to improve vaccine uptake is essential. Passive and active immunoprophylaxis (hepatitis B vaccine and immunoglobulin) and maternal antiviral prophylaxis (MAP) have proven efficacy in preventing the transmission of hepatitis B from mother to child. It is worth noting that infants infected through vertical transmission have a 90% risk of developing chronic hepatitis B infection in adulthood as compared to the 30% in older children who get infected.^6,7^ In 2024, the hepatitis B birth dose vaccine was added to the existing three doses of the hepatitis B vaccine on the EPI calendar in Cameroon although MAP has been available at no cost since 2014. Currently, the vaccine is free for all exposed newborns; however, accessibility in rural areas and home deliveries still remain major challenges.

The overall infant vaccination service in Cameroon is better structured with high uptake than for adults. This has contributed to the high vaccination coverage of three infant doses (97.1%) compared to the adult coverage of 11.8% of the hepatitis B vaccine.^8^ Nonetheless, some health facilities other than the Vaccination Centres, administer the vaccines to persons who buy from pharmacies. Despite the ongoing efforts to eliminate hepatitis B in Cameroon, poor knowledge of the disease and low adult vaccination coverage fuel the spread of the disease within communities.^8^ Studies in other countries like Uganda and Nigeria have reported the urgency of population education, massive screening to identify new cases and improve vaccine uptake.^4,5,9,10^ Therefore, it is imperative to sensitise and test the population towards behaviour change and identification of new cases in the communities. This method was used in Japan for prevention of infection caused by hepatitis viruses.^11^ Also, it was used in Nigeria and Uganda for assessing awareness and for mass screening in selected communities, respectively.^4,10^ Similarly, population sensitisation and screening were key interventions in the fight against the acquired immunodeficiency syndrome (AIDS) pandemic.^12^

We conducted a knowledge, attitude and practice (KAP) and serosurvey in five Health Districts of the Centre and South West Regions in Cameroon on hepatitis B. The objective of this study was to assess KAP, sensitise and identify new cases of hepatitis B in some communities in the two regions of Cameroon.

## Research methods and design

### Study design and duration

A cross-sectional and observational study was conducted in the five Health Districts in the two regions of Cameroon: Buea in the South West Region, Mbankomo, Bikop, Ayos and Ahala in the Centre Region. This study took place from May 2021 to October 2023 in the general population using the convenience sampling method for both KAP and serosurveys.

### Study setting

These communities were both urban (Mbankomo, Ahala and Buea) and rural (Ayos and Bikop). These communities were found in the high-endemic Centre and South West Regions of Cameroon, with a reported pool prevalence in a systematic review of 9.1% and 8.0%, respectively.^13^ The HBsAg rapid diagnostic test (RDT) was used for screening at the voluntary counselling and testing (VCT) health campaign sites, while enzyme-linked immunosorbent assay (ELISA) was used to confirm the reactive cases at the ‘Chantal BIYA’ International Reference Center for research on HIV/AIDS prevention and management (CIRCB) in Yaoundé, Cameroon.

### Study population

All inhabitants living in the 5 Health Districts constituted our study population. Written consents were given separately for the KAP and serosurvey. For the KAP survey, inhabitants of the communities above 21 years who gave consent and those between 15 years and 20 years who gave assent and whose parents or legal guardians gave parental consent were included in the study. On the other hand, for the free hepatitis B screening, all persons living in the communities who gave consent and those below 21 years whose parents or guardians gave parental consent and assent (except for children less than 10 years) were included in the serosurvey.

#### Sampling method

A convenience sampling method was used. These communities were selected based on the ongoing hepatitis B Mother–Infant Cohort (MIC) Hep B Network project.^14^ Two regions were selected to represent two major cultural zones out of the four in Cameroon, the Fang-Betis and the Sawas. The Fang-Betis believe in sexual liberty and regular sex as an essential element in adolescence even among the unmarried, unlike the Sawas, who uphold marriage as a safe space for sexual liberty.^15^ Therefore, some communities in these cultural groups were selected for sensitisation and identification of new cases, through free hepatitis B testing. The trained research team through a door-to-door data collection process, filled the paper-based questionnaire with every eligible inhabitant. On the other hand, all inhabitants in these communities who volunteered to be tested at the outdoor health campaign site and gave consent were included in the study.

The sample size was calculated for the KAP and serosurvey using the formulae below, which gave 130 and 73 participants for the KAP and serosurveys, respectively.^16^

### Sample size calculation for the knowledge, attitude and practice survey


$$\begin{array}{l} n=(Z_{\alpha /2}+Z_{\beta }{)}^{2}*\left(p_{1}(1-p_{1})+p_{2}(1-p_{2})\right)/(p_{1}-p_{2}{)}^{2}\\ n=2(Z_{\alpha /2}+Z_{\beta }{)}^{2}P(1-P)/(p_{1}-p_{2}{)}^{2} \end{array}$$ [Eqn 1]

where *Z*_α__/2_ represents the confidence interval at 95%, which is equal to 5%; *Z*_β_ = *Z*_0.20_ = 0.842 (from *Z* table) at 80% power; *p*_1_ − *p*_2_ = Difference in proportion of events in two groups. *P*_1_ is from our pilot study carried out in the Ayos Health District, which showed an awareness proportion of 21%; *P*_2_ is the 20% improvement from the *P*_1_ that we want to observe (*P*_1_ + 20%) = 41%; *P* = pooled prevalence = (prevalence at baseline [*p*_1_ = 21%] + prevalence at endline [*p*_2_ = 42%])/2 = 31.5%.

### Sample size calculation for the serosurvey


$$n={\left(Z_{\alpha /2}\right)}^{2}P(1-P)/d^{2}$$ [Eqn 2]

where *Z*_α__/2_ represents the confidence interval at 95%; *P* is the estimated prevalence of hepatitis B in Cameroon; *d* is the margin of error of 5%, and *n* is the sample size.

### Data collection

Data were collected in two phases, the first phase was the pre-campaign phase, which consisted of sensitisation and the KAP survey. The second phase was the hepatitis B VCT health campaign to screen inhabitants in these communities who gave informed consent.

#### Pre-campaign period: Sensitisation and knowledge, attitude and practice survey

Community relay agents and/or practising nurses in these communities were trained and evaluated using the sensitisation guide and the paper-based questionnaire before the door-to-door sensitisation and survey. The door-to-door KAP survey started 2 weeks before the VCT health campaign. Eligible persons in the communities who gave written consent or assent and parental consent (only those under 21 years) were administered the questionnaires. Each participant was informed of the importance of confidentiality in responding to the questions in the questionnaire.

Interviews of participants were conducted by trained community relay agents and/or practising nurses in these communities through group or one-on-one interactions using open-ended and closed-ended questions (Online Appendix 1). This questionnaire was tailored from field observations by our research team on the MIC Hep B Network project^14^ and from a study in Uganda.^17^ The questionnaires (in English and French) were interviewer-administered by trained fieldworkers in the participant’s language of preference. The questionnaire was made up of four introductory questions to test awareness. These questions comprised of asking the participants: (1) ‘Have you heard of hepatitis B before this interview?’; (2) ‘Do you know any route of transmission of HBV?’; (3) ‘Do you know the organ affected by the HBV?’ and (4) ‘Do you know a symptom associated with the disease?’. Those who had never heard of the disease did not provide any further answers to the questionnaire, while those who had heard of the disease completed the questionnaire.^17^ The KAP survey questions summed up to 42, categorised as follows: 16 questions on general knowledge of hepatitis B, six questions on knowledge about mother-to-child-transmission (MTCT) of HBV, seven questions on attitudes towards MTCT of HBV and 13 questions on behaviours and practices towards prevention and control of hepatitis B.

After filling out the questionnaire, the sensitisation guide (Online Appendix 2) was used by the interviewer to educate the participants on hepatitis B. In addition, flyers (Online Appendix 3) obtained from the Department of Disease Control and Epidemics in the Ministry of Public Health in Cameroon and the MIC Hep B Network were shared with the participants. Key take-home messages on the prevention of mother-to-child transmission (PMTCT) and horizontal transmission of hepatitis B were highlighted on the flyers.

After the KAP survey, mass sensitisation was carried out in the community through action groups such as schools, churches, hospitals and the City Councils, which gave their authorisation. Announcements of the hepatitis B Sensitisation and Free VCT Campaign were made by both the action groups and the community relay agents using loudspeakers in the community.

### The sensitisation guide

This guide was developed to respond to the frequently asked questions (FAQ) on hepatitis B on the Hepatitis B Foundation website^18^ and from field experiences in the ongoing MICHep B Network project. These tailored responses aimed to meet the educational needs of communities on hepatitis B. It was pre-tested with the community relay agents and practising nurses during the training before it was used for sensitisation in the community (see Online Appendix 2).

#### Voluntary counselling and testing health campaign period

**FIGURE 1:**
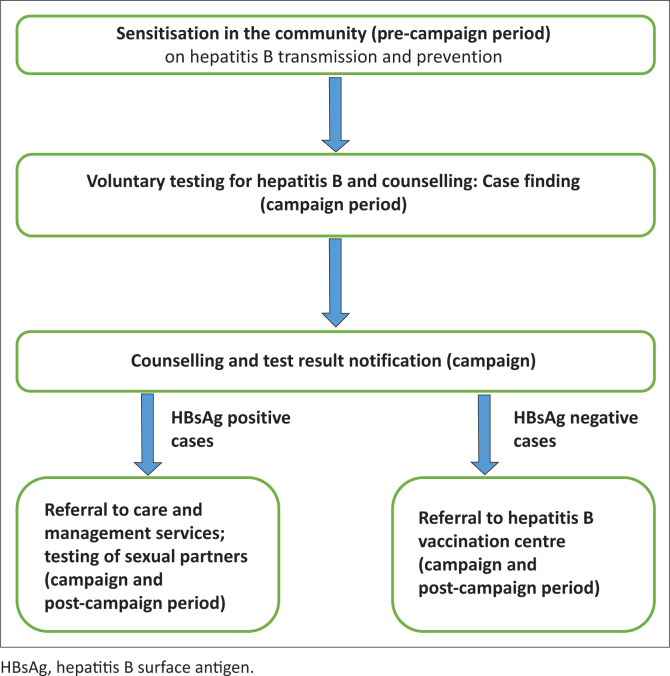
Flow chart of knowledge, attitude and practice and serosurvey data collection.

**Sensitisation, pre-test counselling and free voluntary testing:** The VCT campaigns were organised in four communities (Bikop, Mbankomo, Ahala and Buea). These campaigns were co-facilitated by the Ministry of Health staff and action groups. During the health campaign period, announcements were made in the campaign area and environs to inform the population using loudspeakers. As the population gathered in groups, group education on hepatitis B was followed by question-and-answer sessions. All those who volunteered to be tested signed a written informed consent. Written parental consent and assent (for children above 10 years) were given by the parent or guardian of the participants below 21 years and by the participants, respectively. Each volunteer later benefited from Pre- and Post-test Counselling sessions before result notification while ensuring that stigmatised communication was avoided (Figure 1).

During the health campaign, after the pre-test counselling, enrolment and coding, 5 mL of blood was collected by skilled research staff in ethylene diamine tetraacetic acid (EDTA) tubes from participants. The RDTs were carried out at the campaign site for every participant using whole blood on the ABON™ HBsAg RDTs (RDT, Reference: IHBsg-402, Abbott) to detect the surface antigen of the HBV (HBsAg) following the manufacturer’s instructions. The HBsAg-positive samples were transported to CIRCB in Yaoundé and the Regional Hospital Annex laboratory in Buea using temperature-controlled, leak-proof boxes. The samples were centrifuged and plasma separated and stored at −20 °C. Plasma samples from Buea were transported under the aforementioned conditions to CIRCB in Yaoundé. All plasma samples were analysed using an ELISA Murex HBsAg kit (Reference: 9F80-01/05 GE 34 de DiaSorin S.p.A United Kingdom) by laboratory technicians following the manufacturer’s instructions. The confirmatory results were sent within 2 weeks of collection to the collaborating health facility for the participants.

**Notification of hepatitis B test results and post-test counselling:** Notification of the RDT hepatitis B results and post-test counselling were carried out by a physician in a dedicated space to ensure confidentiality. Irrespective of the results, volunteers were encouraged to bring their partners for testing. Participants who tested negative were encouraged to get vaccinated, and those who tested positive were referred to a hepatitis treatment centre. In addition, participants with difficulties accessing the Vaccination Centers were advised to purchase the hepatitis B vaccine from pharmacies and get vaccinated in an appropriate health facility. Meanwhile, the reactive cases were advised to return for their confirmatory test results (ELISA) within 2 weeks at the health facility.

#### Post-campaign: Care management

The participants who were HBsAg-positive with the confirmatory test were referred to hepatitis treatment centres. The counsellor emphasised partner notification and testing, testing and vaccination of household members and health education on the transmission routes and prevention methods.

### Data analysis

Statistical analysis was conducted using EPI Info version 7.2.6.0. The responses from the questionnaire were ‘Yes’, ‘No’ and ‘I do not know’. Each question with a correct response scored one point, while an incorrect response, ‘I do not know’ or two responses chosen by the participant for the same question scored zero. A cutoff value of 70% was used to grade participants into ‘Favourable’ and ‘Unfavourable’. The cutoffs were 11 out of 16 (70%) for general knowledge on hepatitis B, 5 out of 7 (70%) for attitude, 4 out of 6 (70%) for MTCT knowledge and 9 out of 13 (70%) for practice.^19^ The threshold value for statistical significance of *p*-value was 0.05 and 80% for power. Categorical and qualitative variables were presented as frequencies and proportions, while quantitative variables as means. Univariate and multivariate regression models were used to check for statistical associations between ‘heard of hepatitis B before the interview’, ‘sex’ and ‘level of education’. Moreover, these regression models were used to check for statistical associations among ‘practice’, ‘general knowledge of hepatitis B’, ‘attitude towards MTCT’ and ‘knowledge on MTCT’. The independent variables were ‘level of education’, ‘sex’, ‘general knowledge of hepatitis B’, ‘attitude towards mother-to-child transmission’ and ‘knowledge on MTCT’. In contrast, the dependent variables were ‘heard of hepatitis B before the interview’ and ‘practice’.

### Ethical considerations

This study was carried out in Cameroon, ensuring the respect of all participants’ dignity and rights according to the Helsinki Declaration guidelines for research. The Institutional Review Board (IRB) of the Faculty of Medicine and Biomedical Sciences, University of Yaoundé I in Cameroon approved the implementation of this project with reference numbers, reference number: 490/UYI/FMSB/VDRC/CSD of 2020 and reference number: 0415/UYI/FMSB/VDRC/ DAASR/CSD of 2023.

## Results

### Sociodemographic parameters

A total of 759 participants were recruited, and the mean age was 39.9 ± 12.6 years, ranging from 15 years to 74 years for the KAP survey. The female sex was predominant at 61.4%, and 57.9% of the participants were unmarried (single, cohabiting or divorced), though 61.1% had at least one sexual partner. Moreover, 88.3% were Christians, 49.1% ended their education at the secondary level, and 28.2% were students. The mean age for the serosurvey participants was 34.4 ± 16.9 years, ranging from two to eighty years, and the female sex was predominant with 61.4%.

### Knowledge, attitude and practice survey

#### Awareness

One hundred and fifty-five (20.4%) participants had never heard of hepatitis B and the majority were women, 392 (51.6%). Among the 604 (79.6%) participants who had heard of the disease before the interview and who completed the questionnaires, the following findings were recorded: 315 (52.2%) did not know at least one route of transmission of HBV and 364 (60.3%) were unaware of a symptom associated with the disease. Meanwhile, 314 (52.0%) participants correctly identified the organ affected by the virus.

#### Knowledge, attitude and practice assessment

The 604 participants who had heard of hepatitis B were included in the KAP assessment. Among these participants, 71.0% (*n* = 429/604) and 65.7% (*n* = 397/604) had inadequate general knowledge of hepatitis B and the transmission from mother to child of HBV, respectively. Moreover, 68.7% (*n* = 415/604) had unfavourable practices, while 92.9% (*n* = 561/604) had favourable attitudes towards hepatitis B interventions. In addition, 69.5% (*n* = 420/604) had been tested for hepatitis B before the interview. Furthermore, among the participants, 61.1% (*n* = 245/401) had sexual partners, while 12.7% (*n* = 39/308) knew the hepatitis B status of their partners. An association was observed between knowledge of hepatitis B and practice towards hepatitis B prevention with a crude odds ratio of 4.2, confidence interval [2.8–6.0].

‘Level of education (university)’ had a significant odds ratio of 2.9; therefore a higher likelihood to have heard of hepatitis B. The results also show that the participants with good ‘general knowledge of hepatitis B’ were more likely to practice prevention methods.

**TABLE 1:** Awareness of hepatitis B among participants.

Questions on awareness	Yes			No		
	*N*	*n*	%	*N*	*n*	%
Have you ever heard of hepatitis B before (*n* = 759)?	759	604	79.6	759	155	20.4
Do you know a route of transmission of hepatitis B virus?	604	444	47.8	604	315	52.2
Do you know the organ affected by hepatitis B virus?	604	314	52.0	604	290	48.0
Do you know a symptom associated with hepatitis B?	604	240	39.7	604	364	60.3

**TABLE 2:** Multivariate logistic regression for sociodemographic factors differentiating those who had heard of hepatitis B and those who had never heard.

Predictor	Reference category	Odds ratio	95% confidence interval	*p*
Sex (male)	Female	0.6536	0.4490–0.9516	0.0265
Education level (Primary)	No official education	0.5071	0.2005–1.2825	0.1515
Education level (Secondary)	No official education	0.7886	0.3247–1.9153	0.5999
Education level (University)	No official education	2.9185	1.0913–7.8051	0.0328

Note: Codes used for predictors: male = 1, female = 0; no official education = 0, primary = 1, secondary = 2, university = 3; had never heard of hepatitis B = 0, had heard of hepatitis B = 1.

**TABLE 3:** Multivariate logistic regression for likelihood factors differentiating participants with favourable practice and unfavourable practice.

Predictors	Reference category	Odds ratio	95% confidence interval	*p*
Attitude towards MTCT prevention and control (favourable)	Unfavourable	1.5090	0.9628–2.3651	0.0727
General knowledge on hepatitis B (adequate)	Inadequate	4.1885	2.8378–6.1820	0.0000
Knowledge on mother-to-child transmission (favourable)	Unfavourable	0.7536	0.4878–1.1645	0.2027

Note: Codes used: unfavourable attitude = 0, favourable attitude = 1; inadequate knowledge = 0, adequate knowledge = 1; unfavourable practice = 0, favourable practice = 1.

MTCT, mother-to-child transmission.

#### Vaccination against hepatitis B

Of the 604 participants who had heard of hepatitis B, 56.8% (*n* = 343/604), 53.1% (*n* = 321/604) and 60.1% (*n* = 363/604) of the participants knew of the hepatitis B vaccine, supported the idea that the vaccine be made compulsory and underscored that the vaccine was expensive. In addition, 5.1% (*n* = 39/759) of the participants were reported to be vaccinated.

### Sensitisation and serosurvey

The health campaign was co-facilitated by the Ministry of Public Health staff, faith-based organisations, the City Council, schools (four primary schools and one secondary school), a cultural group and a non-profit organisation. A total of 456 people in the general population gave written consent to be tested for hepatitis B from these communities. The mean age of the volunteers was 34.4 ± 16.9 years. The total number of participants for the serosurvey was 272 and 184 for the Centre and South West Regions. Of note, some parents who came for the screening brought their children later for screening after going through the counselling and testing. The overall proportion of hepatitis B in this study was 8.3% (*n* = 38/456). The proportion of hepatitis B by region was 12.1% (*n* = 33/272) and 2.7% (*n* = 5/184) for the Centre and South West Regions, respectively. Despite the low male proportion of 39.0% (*n* = 178/456), they represented 55.3% (*n* = 21/38) of the HBV-infected persons. All the children below 16 years (*n* = 75) were negative for HBsAg. Of the 199 persons who were asked if they had been tested for HBsAg before the campaign, 81.9% (*n* = 163/199) were unsure or had never been tested. One hundred and ninety-eight (*n* = 198) tested adults were equally interrogated on their vaccination status and 4.5% (*n* = 9/198) were vaccinated. However, infants and adolescents were excluded because most parents could not affirm if they were administered the pentavalent vaccine (hepatitis B vaccine is incorporated in the pentavalent vaccine) in the EPI programme.

## Discussion

This study sought to assess the knowledge, attitude and practice, sensitise the population and identify new cases of hepatitis B. We observed a low awareness of hepatitis B, and about 20% of the participants had never heard of hepatitis B. The proportion of hepatitis B-positive cases was 8.3%. More than 50% of the participants were in favour of compulsory vaccination, but the vaccination coverage was at 5%. In addition to the aim of the study, the project also contributed to training community relay agents and/or practising nurses in the community. This action was targeted at ensuring sustainable sensitisation actions in these communities through community-engaging strategies and action groups. Strong co-facilitation by the Ministry of Public Health staff, faith-based organisations, the City Council, schools, a cultural group and a non-profit organisation enhanced sensitisation and mobilisation of the communities. This co-facilitation also minimised psycho-social barriers encountered in the communities during the project. Moreover, training and recruiting community relay agents facilitated the KAP survey interactions and ensured post-health campaign sustainability. A seroprevalence of 8.3% was observed, with the male population having a higher proportion despite being under-represented in the study. This proportion is higher than those reported in other studies, 6.4% and 7.1% in Cameroon but similar to the 8.7% previously reported in Cameroon and lower than the 13% reported in Chad.^8,20,21,22^ This study was conducted in both rural and urban communities in Cameroon, yet 155 (20.4%) out of 759 participants had never heard of hepatitis B. This finding is lower than the 62.9% obtained in Uganda and 44.2% in Gabon, but higher than the 3.8% reported in Ghana.^17,23,24^ However, a study carried out in Gabon reported that 44.5% of pregnant women had not heard of the disease.^23^

Furthermore, more than half of the participants knew the organ affected by the virus. In contrast, over half of the participants did not know at least one route of transmission of HBV, even though two-thirds were sexually active. This is higher than the findings on HBV transmission routes of 33.5% in Ghana, but similar to the 48.1% reported in Gabon among pregnant women.^23,25^ This underscores the low awareness of the disease in the population but also highlights the need for sensitisation and free screening to curb the transmission chain in these highly endemic communities. Tracking unidentified cases of hepatitis B infections in the community, linking to care and increasing awareness in the communities (Online Appendix 4,^12^ Online Appendix 5) will empower communities to make informed decisions on treatment and prevention.^8^

Low knowledge of hepatitis B infection in a predominantly sexually active population is a substantial drawback to achieving the GHSS on viral hepatitis goals set by WHO.^12^ Although horizontal transmission is common among adults, vertical transmission remains the predominant route of transmission in sub-Saharan Africa that leads to chronicity.^8^ However, more than two-thirds (71.0%) of our participants had inadequate knowledge of the disease, and a majority did not know of HBV MTCT. This proportion (inadequate knowledge) is higher than the 47.1% obtained in Cameroon and 44.2% reported in Gabon in urban settings but similar to the 64.6% reported in Ethiopia.^8,23,26^ This suggests the necessity to scale sensitisation efforts among both rural and urban populations in order to bridge the knowledge gap, which could otherwise hamper the elimination of hepatitis B.

In addition, about two-thirds of the participants had poor knowledge and practices regarding hepatitis B. Most participants (92.7%) had favourable attitudes towards the prevention and treatment of hepatitis B. More so, one-third of our participants had adequate general knowledge of hepatitis B, which is similar to the findings in Ethiopia reported at 26.6%.^18^ These similarities suggest low awareness of hepatitis B in different populations and underscore the necessity of sensitisation, hence empowerment of the population towards behaviour change. Furthermore, the poor practice observed in this study is similar to the findings in the Muyuka and Limbe Health Districts in the South West Region, Cameroon, which reported 68.4% and 63%, respectively, for pregnant women.^27,28^ Despite the favourable attitudes towards PMTCT of HBV, the poor practices observed could be linked to poor knowledge of hepatitis B. This is mirrored by the fact that those who had poor knowledge of the disease were four times more odds to depict poor practices towards prevention and control measures. These observations could be indicative of the need to scale sensitisation in order to empower communities (with knowledge and competence) and therefore improve or change behaviours. Moreover, ‘no parent wants their child sick’ is an unavoidable logical explanation for the favourable attitude towards hepatitis B PMTCT. To that effect, scaling existing efforts by different task forces to improve knowledge and create awareness would impact the practices in these communities towards hepatitis B prevention and treatment.

A low vaccination coverage of 5.1% was observed in the communities even though the seroprevalence was high. Other studies in Cameroon have demonstrated a lower adult vaccination rate of 2.5% in the Centre Region, but higher rates of 11.7% and 11.4% have also been reported in the Centre Region and nationwide, respectively.^9,14,29^ More than half of the participants who had heard of the disease knew of the vaccine and agreed to compulsory vaccination yet were unvaccinated. This suggests the lack of financial accessibility, as approximately two-thirds of the participants reiterated that the adult vaccine was expensive. Furthermore, the subvention of the vaccine to render it more affordable will increase vaccination coverage in these communities. Scaling such health campaigns coupled with other interventions will prevent about 4.5m premature deaths in the low- and middle-income countries, according to WHO, by 2030.^3,12^

Vaccination is the mainstay prevention method towards the global elimination of hepatitis B. Therefore, it is important to sensitise and identify new cases in the community. This could be done through massive free voluntary testing accompanied by pre- and post-test counselling, followed by linkage to treatment and care or referral for vaccination. Above all, this all starts with sensitisation and health education as a bedrock to eliminate hepatitis B. Several hepatitis B elimination policies and interventions already exist but are inadequately implemented in the Cameroonian setting. There is a need to create awareness, increase vaccine uptake, integrate the HBV PMTCT Programme into the well-structured HIV PMTCT Programme and create more treatment centres and accessible vaccination services. Hepatitis B vaccination acceptance was high and the post-sensitisation experience with the participants was characterised by the willingness to get the vaccine, but most reiterated being financially limited. Therefore, strengthening strategies to bridge this gap will have a ripple effect leading to positive advancements towards the hepatitis B elimination goal of 2030.

### Strengths and limitations of the study

This study creates awareness in communities of hepatitis B and further shows the existence of the disease in these communities through VCT health campaigns. The findings of this study will be used to improve the ongoing project. This study did not assess the effect of the intervention on the communities, but it is in prospect for future studies. The convenience sampling method was used because recruited participants were only those who gave consent (within the study period), and no random selection method was used. Also, there was no existing community map of the households in these communities, which made random selection difficult. However, announcements were made to encourage the community to participate massively in the mass hepatitis B screening campaign, while the door-to-door KAP survey included all consenting household participants in these communities. The data collection through questionnaires was subjective; therefore, these findings should be interpreted with caution.

### Recommendation

Hepatitis B is a silent killer that exists in our communities, even though the population remains unaware. Health promotion actions such as sensitisation and VCT health campaigns will create awareness and promote prevention interventions such as vaccination and treatment. More studies will be carried out to evaluate the post-intervention (VCT health campaign) impact on awareness and practice.

## Conclusion

We observed low awareness of hepatitis B and a high seroprevalence of HBsAg. Low knowledge of the disease enhances unfavourable practices towards hepatitis B prevention and control. Therefore, community-engaging interventions to mitigate the spread of the disease through sensitisation and VCT health campaigns will bridge the knowledge gap and improve behaviours.
